# GCUNet: a graph neural network-based contextual learning network for tertiary lymphoid structure semantic segmentation in whole slide image

**DOI:** 10.1186/s42492-026-00227-z

**Published:** 2026-07-31

**Authors:** Lei Su, Ruiyu Li, Guangyao Zhang, Zonghao Liu, Xuqing Geng, Hetong Wang, Weihao Gai, Huanwen Wu, Jie Tian, Yang Du

**Affiliations:** 1 CASMI, Institute of Automation, Chinese Academy of Sciences, Beijing 100190, China; 2 School of Artificial Intelligence, University of Chinese Academy of Sciences, Beijing 100049, China; 3 Department of Pathology, Molecular Pathology Research Center, Peking Union Medical College Hospital, Chinese Academy of Medical Sciences and Peking Union Medical College, Beijing 100730, China; 4 Clinical Oncology School, Fujian Medical University, Fujian Cancer Hospital, Fuzhou, Fujian 350014 China; 5 National School of Elite Engineering, University of Science and Technology, Beijing 100083, China

**Keywords:** Tertiary lymphoid structure, Semantic segmentation, Whole slide image, Graph neural network, Contextual learning

## Abstract

This study focuses on tertiary lymphoid structure (TLS) semantic segmentation in whole slide images (WSIs). Unlike TLS binary segmentation, TLS semantic segmentation identifies boundaries and maturity and requires the integration of contextual information to discover discriminative features. Owing to the extensive scale of WSI (e.g., 100,000 *×* 100,000 pixels), TLS segmentation is typically performed using a patch-based strategy. However, this prevents the model from accessing information outside the patches, thereby limiting its performance. To address this issue, GCUNet, a graph neural network-based contextual learning network for TLS semantic segmentation, is proposed. Given an image patch (target) to be segmented, GCUNet first progressively aggregates the long-range and fine-grained contexts outside the target. Subsequently, a detail and context fusion block (DCFusion) was designed to integrate the context and details of the target to predict the segmentation mask. This study builds four TLS semantic segmentation datasets: TCGA-COAD, TCGA-LUSC, TCGA-BLCA, and PUMCH-PAAD. The first three, comprising 826 WSIs and 15,276 TLSs, will be made publicly available to promote TLS semantic segmentation. Experiments on these datasets demonstrate that GCUNet consistently improves the mean F1-score (mF1) performance compared with existing state-of-the-art methods, with an observed mF1 improvement of at least 7.41% over the best-performing baseline. These results highlight the potential of GCUNet for accurate TLS assessment and facilitate the development of computational pathology-based immune microenvironment analysis.

## Introduction

**Fig. 1:**
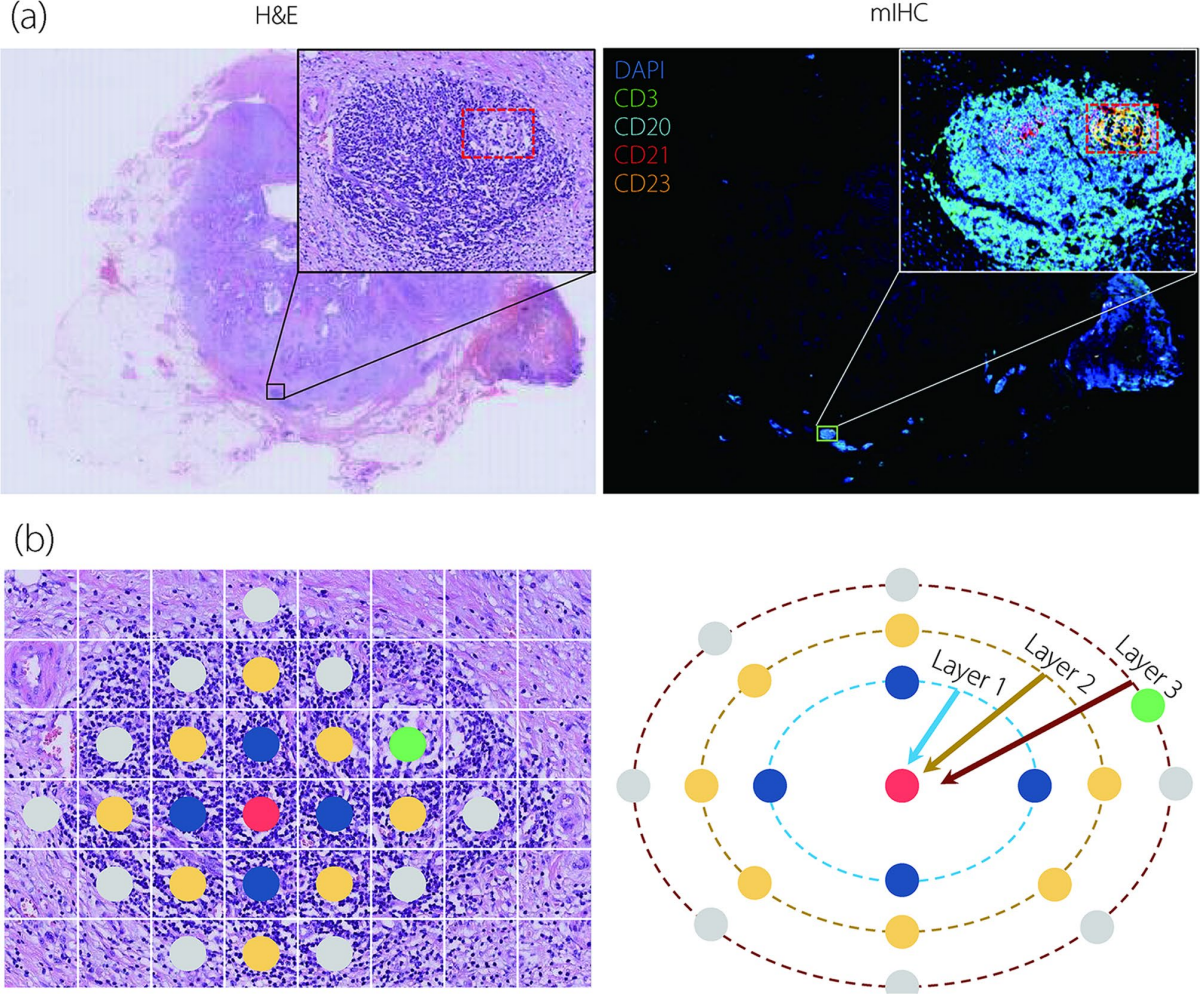
Gcunet gathers discriminative features by aggregating contextual information outside the target patch. (**a**) Comparison of SEL-TLS in H&E and mIHC. The SEL-TLS contains a germinal center (GC), which is highlighted by the red box. In H&E, a pale staining region represents GC. In mIHC, the GC is identified by CD23+. The mIHC includes DAPI (deep blue, for nucleus), CD3 (green, for T cells), CD20 (light blue, for B cells), CD21 (red, for follicular den-dritic cells), and CD23 (orange, for GC). (**b**) Left. the SEL-TLS is divided into multiple patches for segmentation. The red node represents the target patch for segmentation, the blue nodes are first-order neighbors, the yellow nodes are second-order neighbors, and the gray and green (GC, which determines TLS maturity) nodes are third-order neighbors. right. GCUNet progressively aggregates contextual information outside the target patch by graph convo-lutional network (GCN) layers

Tertiary lymphoid structure (TLS) is an aggregate of immune cells that can be classified into three levels of maturity: early TLS (E-TLS), primary follicle-like TLS (PFL-TLS) and secondary follicle-like TLS (SFL-TLS) [1, 2]. In most solid tumors, the presence of TLS is closely associated with the anti-tumor immune response, which is significantly influenced by the maturity of TLS. To identify the maturity levels of TLS, multiplex-immunofluorescence (mIF) is commonly used to detect specific molecular expressions, such as CD21${}^+$ for PFL-TLS and CD23${}^+$ for SFL-TLS. However, the widespread adoption of this approach is limited by time and economic costs as well as the available examination techniques. Fortunately, molecular expression leads to morphological changes in the nucleus and tissue structures [3, 4]. As shown in Fig. 1a, SFL-TLS not only expresses CD23${}^+$ but also exhibits a germinal center (GC) in a whole slide image (WSI) stained with hematoxylin and eosin (H&E). Therefore, the identification of TLS in WSI is important for tumor diagnosis and treatment.

In recent years, computational pathology (CPath) [5] has attracted increasing attention because of its wide range of applications, including cancer classification [6–8], tumor grading [9, 10], survival analysis [11–13], and biomarker prediction [14–16]. Binary segmentation is a critical approach for delineating boundaries of the TLS [17–21]. Owing to the extensive scale of WSI (e.g., 100,000 *×* 100,000 pixels), the segmentation procedure is often performed in two steps: First, the WSI was divided into numerous image patches, and each patch was processed using a segmentation model. Second, the patch-level results were assembled into the entire TLS segmentation image. However, for the TLS semantic segmentation task, this approach lacks awareness of contextual information beyond the target patch, which restricts its ability to uncover discriminative features, thereby limiting segmentation performance.

In WSI analysis, contextual learning methods based on convolutional neural networks (CNNs) [20–25], transformers [7, 26, 27], and graph neural networks (GNNs) [16, 28–32] have been developed to capture multi-scale contextual information or enhance global context awareness.

However, prior work has primarily focused on WSI-level tasks [26, 28, 30, 32] and patch-level tasks [7, 25, 29, 31], such as survival risk prediction and patch classification. Existing methods for pixel-level tasks [20–24] relied exclusively on CNNs and low-resolution images, limiting their ability to capture long-range and fine-grained contextual information. Applying contextual learning to pixel-level segmentation tasks in WSI, such as TLS semantic segmentation, remains an area worthy of further exploration.

To address this issue, a GNN-based contextual learning network (GCUNet) is proposed for capturing long-range and fine-grained contextual information outside the target patch. As illustrated in Fig. 1b, the model progressively aggregates contextual information outside the target. Additionally, a detail and context fusion block (DCFusion) was designed to perform the semantic-level fusion of contextual and detailed information. Four cancer-type TLS semantic segmentation datasets were built and it was demonstrated that GCUNet achieves a higher segmentation performance than the compared methods, achieving at least a 7.41% improvement in mean F1-score (mF1) over state-of-the-art methods (SOTA). The main contributions of the proposed method are as follows:

- This study addresses a novel task: TLS semantic segmentation in WSI. To the best of the authors’ knowledge, this work represents the first approach to capture contextual information outside of the target patch for TLS semantic segmentation.
- A new GNN-based contextual learning method GCUNet for TLS semantic segmentation is presented. GCUNet leverages GCNs to flexibly aggregate long-range and fine-grained contextual information outside patches, while the designed DCFusion performs a semantic-level fusion of detailed and contextual information to predict segmentation masks.
- Four datasets are gathered from different cancer types for validation. Considering the difficulty of acquiring pixel-level annotations in WSI, Three annotated datasets based on TCGA (TCGA-COAD, TCGA-LUSC, TCGA-BLCA, comprising 826 WSIs and 15,276 TLSs) will be released to promote TLS semantic segmentation.

### TLS segmentation in WSI

Three maturity stages of TLS are classified based on the presence of follicular dendritic cells or GC [33]. Existing end-to-end methods for TLS segmentation primarily outline the boundaries without assessing the maturity. Barmpoutis et al. [17] used a segmentation model with dilated convolutions for the binary segmentation of TLS and refined the boundaries using an active contour model. Wang et al. [18] introduced a CNN-based model for segmenting TLS boundaries from WSI to compute prognostic biomarkers. Chen et al. [19] proposed a model that simultaneously segmented TLS, lymphocytes, and tissue foreground for prognostic analysis of various cancer types. van Rijthoven et al. [20] utilized a multi-resolution model to segment TLS from low-resolution images, capturing both coarse-grained and short-range contextual information. In 2024, van Rijthoven et al. [21] employed three datasets to perform binary segmentation of TLS. Given the different values of the three maturity levels, Li et al. [34] classified TLS into one of three grades based on the features of the lymphocyte density map. Unlike previous studies, an end-to-end TLS segmentation model is proposed, to achieve segmentation of TLS across the three maturity stages, defining this task as TLS semantic segmentation.

### Contextual learning for segmentation in WSI

Considering WSI with pyramidal resolutions, researchers typically use networks with low-resolution branches to capture the context of a target patch. Gu et al. [22] first introduced a low-resolution channel into U-Net [35] to encode contextual information and guide the encoding and decoding processes of the network. Ho et al. [23] developed a deep multi-resolution network with multiple encoder-decoder branches to extract more comprehensive contextual information. To ensure pixel-level spatial alignment of the details and context of the target patch, Schmitz et al. [24] integrated CNN segmentation networks at different scales to incorporate contextual information across various resolutions in a segmentation task. van Rijthoven et al. [20] aligned feature maps at the same resolution using the *Hooking* mechanism. Although the aforementioned methods emphasize the importance of contextual information, more effective approaches to learning and integrating contextual information are still being explored.

Transformer-based architectures have also been widely investigated for medical-image segmentation. TransUNet [36] combines convolutional feature extraction with a vision transformer encoder to model long-range dependencies, while preserving fine-grained spatial information through U-shaped skip connections. Swin-UNet [37] adopts a hierarchical encoder—decoder transformer based on window and shifted-window self-attention, enabling local and cross-window feature interactions with reduced computational complexity. H2Former [38] integrates hierarchical CNN features, multiscale channel attention, and transformer-based global modeling to improve local—global feature representation. DTMFormer [39] introduced dynamic token merging to reduce redundant tokens and improve the efficiency of long-range dependency modeling.

Although these approaches enhance contextual representation within an input image or feature map, their accessible context remains limited to the tokens derived from the current input patch when applied to patch-based WSI segmentation. Consequently, they cannot explicitly incorporate the tissue structures located outside the target patch. By contrast, GCUNet constructs a spatial graph over foreground patches and progressively aggregates contextual information from neighboring patches through graph convolution. The resulting external contextual representation is then integrated with the fine-grained features of the target patch using DCFusion. Therefore, GCUNet complements intrapatch transformer modeling by explicitly introducing interpatch contextual reasoning for WSI segmentation.

### GNN-based contextual learning in WSI

In WSI analysis, GNNs are commonly used to model the contextual information between patches or cells, thereby enabling the performance of tasks at either the WSI or patch level. Lu et al. [16] constructed a cell graph to capture global contextual information for biomarker prediction in breast cancer. Chen et al. [12] used GCN to learn the global contexts of WSI for survival predictions. Hou et al. [28] introduced a heterogeneous graph to learn multiscale contextual information for tumor typing and staging. Shi et al. [32] investigated a cross-scale spatial context based on a hierarchical graph for pathological primary tumor staging. Zheng et al. [40] used dynamic graphs to describe the flexible interaction between patches in WSI for tumor typing and staging. In addition to WSI-level tasks, GNN-based contextual learning has been applied to WSI classification tasks at the patch level [31]. Unlike these methods, the proposed model leverages GNNs to capture contextual information outside the target patch for pixel-level tasks.

## Methods

### Pipeline overview

**Fig. 2:**
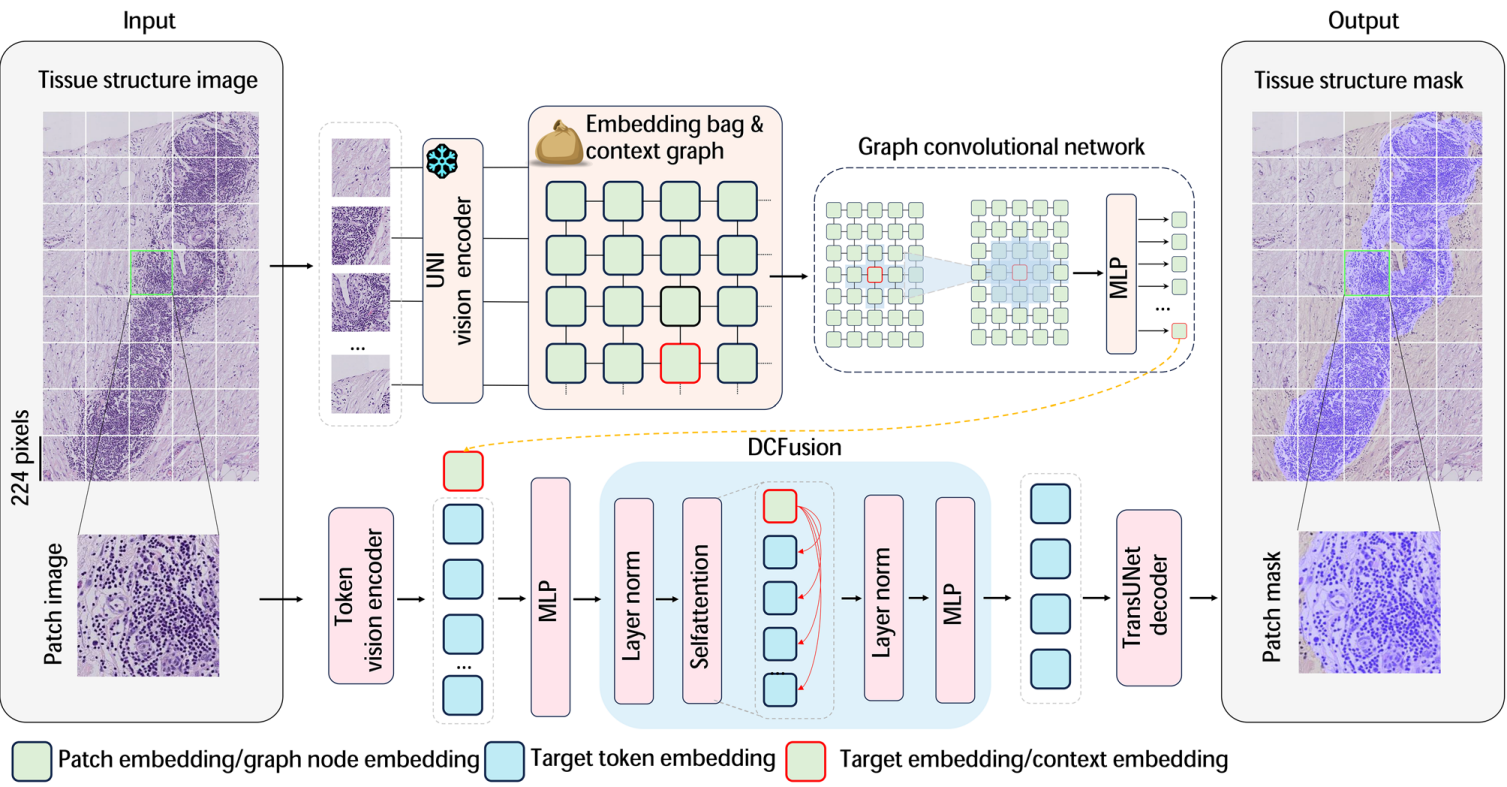
Architecture of GCUNet. In the contextual branch, foreground patches are encoded by a frozen UNI and organized into a spatial context graph. For each target patch, the graph convolutional network (GCN) progressively aggregates information from multi-hop neighboring patches to generate an external contextual embedding. In the segmentation branch, the target patch is independently processed by the encoder of TransUNet to extract fine-grained token representations. detail and context fusion (DCFusion) appends the GCN-derived contextual embedding to the TransUNet target tokens and jointly models them through self-attention and multilayer perceptron blocks. The fused target tokens are subsequently decoded by the TransUNet decoder to predict the patch-level segmentation mask, and the patch predictions are assembled to produce the whole slide segmentation result

The semantic segmentation of TLS aims to delineate the boundaries of TLS at different maturation stages (E-TLS, PFL-TLS, and SFL-TLS) from WSI. In this process, a TLS may be divided into multiple patches, some of which contain significant discriminative information (e.g., GC) that determines the maturation level of the TLS. Therefore, given an image patch (target) to be segmented, the model must be aware of the contextual information outside the target patch to discover significant discriminative features. The proposed method consists of two steps. First, the multi-layer GCN iteratively aggregates long-range and fine-grained contextual information outside the target patch. Then, DCFusion integrates the contextual and detailed features of the target patch at the semantic level to predict the segmentation mask. The proposed GCUNet architecture is shown in Fig. 2.

### Context graph construction

To model the contextual relationships of all patches in WSI, a context graph $\boldsymbol{G} = (\boldsymbol{V}, \boldsymbol{E})$ was constructed where $\boldsymbol{V}$ denotes the set of patch features and $\boldsymbol{E}$ represents the set of edges that connect patches. Specifically, the foreground of WSI was filtered following [41] and divided into non-overlapping patches, resulting in a set of * N * image patches $\boldsymbol{P} = \{\boldsymbol{p}_i \mid i=1 \ldots N \}$. UNI [42] was used to encode each $\boldsymbol{p}_i$ into a feature vector $\boldsymbol{v}_i \in \mathbb{R}^{1024}.$ UNI is a transformer-based vision encoder for CPath that is pretrained on millions of pathology images using a self-supervised method. Consequently, a WSI is represented as a set of * N * nodes $\boldsymbol{V} = \{\boldsymbol{v}_i \mid i=1 \ldots N \}$. Next, the undirected edge set $\boldsymbol{E} = \{\boldsymbol{v}_i \boldsymbol{v}_j \mid (i,j) \in \mathcal{H} \}$ for the graph is determined based on the spatial connectivity of the patches, where $\mathcal{H}$ represents the set of naturally connected nodes using 4-connectivity.

### Contextual information aggregation

A context graph models the features and contextual relationships of each patch. The adjacency matrix $A = [a_{ij}]_{n \times n}$ is derived from the relationship between the graph nodes. The elements of the adjacency matrix are defined as follows:

1 $$a_{ij} = \begin{cases} 1, & \text{if } [\boldsymbol{v}_i, \boldsymbol{v}_j] \in \boldsymbol{E} \\0, & otherwise\end{cases}$$

where the feature matrix $\boldsymbol{X}^{(0)}$ $= \{\boldsymbol{x}_1^{(0)}, \boldsymbol{x}_2^{(0)}, \dots, \boldsymbol{x}_N^{(0)} \}$ $\in \mathbb{R}^{N \times 1024}$ represents the initial feature map for the * N * nodes, with each $\boldsymbol{x}_i^{(0)} = \boldsymbol{v}_i.$ The aggregation of contextual information outside the patches over *t* steps can be expressed as:

2 $$\boldsymbol{X}^{(t)} = \mathrm{F}_{\mathrm{GCN}}(\boldsymbol{X}^{(t-1)}) = \sigma(\tilde{\boldsymbol{A}} \boldsymbol{X}^{(t-1)} \boldsymbol{W}^{(t-1)})$$

where $\tilde{\boldsymbol{A}} = \boldsymbol{D}^{-1/2}(\boldsymbol{A} + \boldsymbol{I})\boldsymbol{D}^{-1/2}$ represents the normalized adjacency matrix, which is computed to balance the number of neighbors for each node, and $\boldsymbol{D}$ denotes the degree matrix. The target node $x_i$ aggregates features from its neighbors, progressively expanding the scope of its contextual information.

After $T_0$ aggregation steps, the feature of node * i * is updated from $\boldsymbol{x}_i^{(0)}$ to $\boldsymbol{x}_i^{(T_0)}$, incorporating contextual information from increasingly distant neighbors. The set of neighbors within * t * steps from *i*-th node is denoted as $\mathrm{Nera}_t(\boldsymbol{x}_i) = \{\boldsymbol{x}_j \mid d(i,j) = t \}$, where * d(i,j) * represents the shortest path length between *i*-th node and *j*-th node. Therefore, the features of the *i*-th node is updated based on the union of features from all neighbors up to $T_0$ steps, which can be expressed as follows:

3 $$\boldsymbol{x}_i^{(T_0)} = \mathrm{F}_{\mathrm{GCN}}^*(\bigcup_{t=1}^{T_0} \mathrm{Nera}_t(\boldsymbol{x}_i^{(0)}))$$

where $\mathrm{F}_{\mathrm{GCN}}^*$ is the graph convolution operation that updates the features of the node * i * by aggregating the information from its neighbors at increasing hops. This process enables the feature of the node to capture long-range contextual information and ultimately learn a richer representation of the target patch. Consequently, multiple GCN aggregation steps enable $\boldsymbol{v}_i$ to learn the increasingly distant contextual information.

### Fusion of detail and contextual information

As illustrated in Fig. 2, after obtaining the long-range contextual features $\boldsymbol{z}_i^c = \boldsymbol{x}_i^{(T_0)} \in \mathbb{R}^{1 \times L}$ for the image patch $\boldsymbol{p}_i \in \mathbb{R}^{H \times W \times 3}$, the encoder from TransUNet [36] was utilized to extract the detailed features $\boldsymbol{z}_i^d \in \mathbb{R}^{b^2 \times L}$, where $b^2 = \frac{HW}{l^2}$ represents the number of tokens for the image patch $p_i$.

Before the detailed features $\boldsymbol{z}_i^d$ and the long-range contextual features $\boldsymbol{z}_i^c$ are fed into the DCFusion module for fusion, positional encoding is applied to the contextual features $\boldsymbol{z}_i^c$. The two feature types are then concatenated into $\boldsymbol{z}_i^{(0)} = [\boldsymbol{z}_i^c + \boldsymbol{e}_{\mathrm{pos}}; \boldsymbol{z}_i^d] \in \mathbb{R}^{(b^2 + 1) \times L}$. The distinctiveness of the overall context in the contextual features $\boldsymbol{z}_i^c$ was enhanced by the addition of positional encoding.

DCFusion consists of * ℓ * layers of multi-head attention and a fully connected block. The final fused features are computed as follows:

4 $$\begin{aligned}\hat{\mathbf{z}}_i^{(l)} &= \operatorname{MSA} \left( \operatorname{LayerNorm}\left(\mathbf{z}_i^{(l-1)}\right)\right)+\mathbf{z}_i^{(l-1)}\\\mathbf{z}_i^{(l)}&=\operatorname{MLP}\left(\operatorname{LayerNorm}\left(\hat{\mathbf{z}}_i^{(l)}\right)\right)+\hat{\mathbf{z}}_i^{(l)}\end{aligned}$$

where $\hat{\mathbf{z}}_i^{(l)}$ denotes the intermediate representation produced by the self-attention sublayer, and $\mathbf{z}_i^{(l)}$ denotes the output of the subsequent MLP sublayer. The MLP is a feed-forward block used to further transform the fused token representations.

### Decoding fused features for segmentation

After obtaining the fused features $\boldsymbol{z}_i^{(\ell)}$ that integrate both the detailed and contextual information of $p_i$, the token features and contextual information within $\boldsymbol{z}_i^{(\ell)}$ were assumed to be fully integrated. Therefore, only the features corresponding to the token positions, denoted as $\boldsymbol{z}_i^{\prime (\ell)} \in \mathbb{R}^{b^2 \times L}$ were selected. These features are then fed into the decoder of TransUNet [36] to predict the segmentation mask $\boldsymbol{y}_m^{\mathrm{pred}} \in \mathbb{R}^{k \times H \times W}$, where *k* refers to a predefined number of categories for the segmentation targets. Finally, the segmentation loss was computed using cross-entropy, and the network was optimized through backpropagation.

5 $$L_{\mathrm{CE}} = -\frac{1}{HW} \sum_{h=1}^{H} \sum_{w=1}^{W} \sum_{c=1}^{K} y_{c,h,w}^{\mathrm{target}} \log \left( y_{c,h,w}^{\mathrm{pred}}\right]$$

The trainable components of GCUNet are jointly optimized, while the UNI encoder remains frozen.

### Datasets

For the pancreatic adenocarcinoma (PUMCH-PAAD) dataset, two adjacent tissue sections from each patient were selected: one for H&E staining and the other for mIF staining, including CD3, CD20, CD21, and CD23. Aided by mIF, the pathologists annotated the boundaries and classified them into three maturation stages in WSI. For the Cancer Genome Atlas (TCGA) colon adenocarcinoma (TCGA-COAD) dataset, the H&E data were downloaded from TCGA and cleaned by excluding low-quality WSI such as those containing artifacts, folds, or large areas of necrosis. TLSs at the three maturity levels were annotated by pathologists without mIF assistance. For the bladder urothelial carcinoma (TCGA-BLCA) and lung squamous cell carcinoma (TCGA-LUSC) datasets, public annotations [21] that highlighted GC and TLS without distinguishing between the maturation stages of the TLS were used. To prepare these data for TLS semantic segmentation task, WSIs that did not contain TLS and classified the TLS into SFL-TLS and non-SFL-TLS (NSFL-TLS) based on the presence of GC were excluded. NSFL-TLS does not contain GC and cannot be distinguished as either E-TLS or PFL-TLS. Therefore, TLS in the TCGA-BLCA and TCGA-LUSC datasets was divided into two categories: SFL-TLS and NSFL-TLS. Finally, the TLS semantic segmentation datasets for the four types of cancer were collected. Two tasks were performed for the four datasets: four-class semantic segmentation (Seg4) and three-class semantic segmentation (Seg3). The PUMCH-PAAD and TCGA-COAD datasets were used for Seg4, entailing semantic segmentation into four categories: background (BG), E-TLS, PFL-TLS and SFL-TLS. TCGA-BLCA and TCGA-LUSC datasets were utilized for Seg3, with categories designated as BG, NSFL-TLS, and SFL-TLS. The detailed statistics of all datasets used in this study are summarized in Table 1. For each dataset, the data into training, validation, and testing sets were randomly divided in a ratio of 6:2:2. The datasets were split at the WSI level to prevent data leakage.

**Table 1:** Overview of dataset count

Task	Data	WSI	E-TLS	PFL-TLS	SFL-TLS	NSFL-TLS	Total
Seg4	PUMCH-PAAD	108	2339	1586	611	–	4536
	TCGA-COAD	225	3496	1034	511	–	5041
Seg3	TCGA-BLCA	342	–	–	498	2538	3036
	TCGA-LUSC	259	–	–	511	6688	7199

WSI: Whole slide image; E-TLS: Early tertiary lymphoid structure; PFL-TLS: Primary follicle-like tertiary lymphoid structure; SFL-TLS: Secondary follicle-like tertiary lymphoid structure; NSFL-TLS: Non-secondary follicle-like tertiary lymphoid structure

### Implementation details

To evaluate the effectiveness of the proposed GCUNet, it was compared with several models based on CNN, Transformer, and multi-resolution approaches, including U-Net [35], attention-UNet [43], Swin-UNet [37], TransUNet [36], H2Former [38], DTMFormer [39], and HookNet [20]. TransUNet was used as a baseline. All competing segmentation models, including U-Net, attention-UNet, Swin-UNet, TransUNet, H2Former, DTMFormer, and HookNet, were trained from scratch without loading external pretrained weights. Their parameters were initialized using the default or architecture-specific random initialization implemented in the corresponding model code. The TransUNet segmentation backbone in GCUNet was initialized using the same settings as in the standalone TransUNet baseline. UNI was not used to initialize, replace, or modify the segmentation backbone. Instead, a pretrained UNI encoder was used exclusively to pre-extract fixed patch-level embeddings for the external contextual branch. The UNI encoder was not included in the segmentation training graph, and its parameters were not updated during optimization.

OTSU was applied [41] to distinguish the foreground region. In most of the experiments, the patch size was set to 224*×*224 with a spatial resolution of 1 *μ*m/pixel. For HookNet [20], the image patch size was set to 256*×*256 to ensure full alignment of feature maps with different resolutions. Softmax was employed in the aggregation function of the GCN layer, with the temperature constant initialized as a learnable parameter set to 1. The hidden feature dimensions were set to 128. A 12-layer attention network with 12 attention heads per layer and a hidden feature dimension of 768 was employed. The fused representations are progressively upsampled using the TransUNet decoder [36], and the output channels are adjusted according to the number of classes in the Seg4 or Seg3 tasks. The batch size was set to 16 and the learning rate was set to $5\times10^{-5}$. In the experiment, we report the F1 score, IoU, precision, and recall for each category. The average values of these metrics across the categories, denoted as mF1, mean intersection over union (mIoU), mP, and mR, were used to evaluate the overall segmentation performance.

The input image was encoded through convolutional layers with parameter normalization, followed by three residual blocks that progressively reduced the feature map size of the target patch by half. In the decoder stage, the fused feature map was scaled up by factors of four, eight, and 16. Finally, the number of output channels in the model corresponded to the number of classes in Seg4 or Seg3.

The model was trained for 100 epochs. AdamW was used to optimize the model with a weight decay of $5\times{10}^{-6}$. The learning rate decay strategy was based on the validation set performance, adjusting the learning rate automatically according to mF1. Specifically, the learning rate was reduced by a factor of 0.95 when mF1 on the validation set did not improve for five consecutive epochs after two cooling periods. To prevent overfitting, an early stopping mechanism based on mF1 with a patience of 50 was employed. Training was halted if the validation performance did not improve for 50 consecutive evaluations. In addition, random data augmentation methods were applied to the training set, including color jitter and Gaussian blur. The experiments were conducted using a PyTorch workstation equipped with a GeForce RTX 4090 GPU. Each GCN layer consists of two convolutional layers with layer-normalization mechanisms. A dropout rate of 0.1 was applied to improve model generalization. Gradient checkpointing was applied to selected GCN layers during training to reduce the activation-memory consumption of the graph-based contextual aggregation module.          

### Comparisons with SOTA methods

Table 2 presents a performance comparison between GCUNet and other methods on the Seg4. GCUNet consistently achieves improved performance across all four evaluation metrics. The UNI pretrained pathology vision encoder is used exclusively as a fixed feature extractor in the external contextual branch to generate patch-level contextual embeddings. It is not used as part of the segmentation backbone, which is based on TransUNet for all experiments. Compared to the baseline, GCUNet improved mF1 by 0.068 and 0.105, representing increases of 12.25% and 18.75% in PUMCH-PAAD and TCGA-COAD, respectively. Notably, the multiresolution network HookNet exhibited suboptimal performance. HookNet improves mF1 by 0.021 and 0.041 compared to U-Net and outperforms the Trans series methods on the two datasets. These results highlight the importance of leveraging contextual information outside the target patch in TLS semantic segmentation. Simultaneously, GCUNet achieves a 7.41% improvement in mF1 over HookNet. These results suggest that long-range and fine-grained contextual information contribute to the observed performance improvement. In the TCGA-COAD dataset, GCUNet outperformed the second-best model, HookNet, with improvements of 11.39 GCUNet achieves consistently higher performance than all compared methods on the Seg4 task.

**Table 2:** The performance of the proposed method and the state-of-the-art on the Seg4 task using the PUMCH-PAAD and TCGA-COAD datasets

Type	Method	PUMCH-PAAD				TCGA-COAD			
		mF1	mIoU	mP	mR	mF1	mIoU	mP	mR
CNN	U-Net [35]	0.559	0.418	0.559	0.563	0.556	0.423	0.557	0.555
	Attention-UNet [43]	0.536	0.399	0.550	0.536	0.528	0.398	0.547	0.547
Trans	Swin-UNet [37]	0.558	0.416	0.562	0.569	0.556	0.424	0.567	0.551
	H2Former [38]	0.543	0.394	0.542	0.525	0.523	0.429	0.429	0.431
	DTMFormer [39]	0.531	0.394	0.539	0.528	0.543	0.413	0.544	0.543
	TransUNet (baseline) [36]	0.555	0.415	0.555	0.556	0.560	0.427	0.575	0.559
Multi-res	HookNet [20]	0.580	0.427	0.608	0.570	0.597	0.463	0.603	0.593
GNN	GCUNet (ours)	**0.623**	**0.474**	**0.655**	**0.613**	**0.665**	**0.523**	**0.676**	**0.660**

The bold values represent the highest scores. mF1: Mean F1-score; mIoU: Mean intersection over union

Table 3 displays the comparative performance of GCUNet against other models for the Seg3 task. The Seg3 task involves three categories: BG, SFL-TLS, and NSFL-TLS. Compared to Seg4, Seg3 is less challenging, resulting in an enhanced overall performance for all evaluated methods. GCUNet achieves the highest mF1 scores on the TCGA-BLCA and TCGA-LUSC datasets, reaching 0.740 and 0.703, respectively. GCUNet improved mF1 by 0.132 compared with the baseline. Meanwhile, GCUNet achieves the best performance on the TCGA-LUSC dataset, where mF1 and mIoU improved by 0.158 and 0.105, respectively, over the baseline. HookNet retained its position as the second-best model on the TCGA-BLCA dataset. However, its lead was considerably reduced, with an mF1 score only marginally higher by 0.009 compared to that of U-Net. For TCGA-LUSC dataset, the second-best model was H2Former. The experiments indicate that the advantage of HookNet diminishes when fewer TLS semantic categories are considered.

**Table 3:** Comparison of segmentation results for the proposed model with other models on the Seg3 task using the TCGA-BLCA and TCGA-LUSC datasets

Type	Method	TCGA-BLCA				TCGA-LUSC			
		mF1	mIoU	mP	mR	mF1	mIoU	mP	mR
CNN	U-Net [35]	0.620	0.486	0.617	0.626	0.546	0.472	0.524	0.572
	Attention-UNet [43]	0.590	0.468	0.616	0.592	0.535	0.458	0.514	0.560
Trans	Swin-UNet [37]	0.620	0.484	0.628	0.626	0.548	0.472	0.614	0.564
	H2Former [38]	0.621	0.488	0.619	0.624	0.566	0.473	0.579	0.568
	DTMFormer [39]	0.566	0.440	0.562	0.572	0.556	0.457	0.555	0.558
	TransUNet (baseline) [36]	0.608	0.480	0.618	0.602	0.545	0.471	0.522	0.575
Multi-res	HookNet [20]	0.629	0.500	0.666	0.608	0.549	0.476	0.552	0.570
GNN	GCUNet (ours)	**0.740**	**0.602**	**0.744**	**0.744**	**0.703**	**0.576**	**0.705**	**0.702**

The bold values represent the highest scores. mF1: Mean F1-score; mIoU: Mean intersection over union

To analyze the factors contributing to the performance improvement of GCUNet, the mF1 scores for BG, E-TLS, PFL-TLS, and SFL-TLS are discussed in Seg4.

As shown in Table 4, GCUNet achieves higher F1 scores for all three TLS maturity categories on both datasets. The proposed model identifies key features by capturing both fine-grained and long-range contextual information beyond the target patch. HookNet lacks robustness in distinguishing the background, resulting in two extreme outcomes on PUMCH-PAAD and TCGA-COAD. This suggests that TLS semantic segmentation performance is hindered by the lack of contextual information outside the target patch. The improvement in model performance was primarily due to the ability to distinguish between the three maturity levels and the long-range, fine-grained contextual information provided by GNNs.

**Table 4:** Comparison of F1 for GCUNet and other models on the PUMCH-PAAD and TCGA-COAD datasets

Type	Method	PUMCH-PAAD				TCGA-COAD			
		BG	E-TLS	PFL-TLS	SFL-TLS	BG	E-TLS	PFL-TLS	SFL-TLS
CNN	U-Net [35]	0.889	0.444	0.409	0.496	0.930	0.426	0.415	0.451
	Attention-UNet [43]	0.889	0.348	0.472	0.433	0.913	0.429	0.350	0.419
Trans	Swin-UNet [37]	0.882	0.455	0.381	0.514	0.929	0.437	0.443	0.417
	H2Former [38]	0.888	0.370	0.406	0.458	0.929	0.429	0.431	0.383
	DTMFormer [39]	0.886	0.401	0.425	0.413	0.911	0.416	0.469	0.397
	TransUNet (baseline) [36]	0.888	0.418	0.408	0.506	0.930	0.452	0.461	0.339
Multi-res	HookNet [20]	0.837	0.483	0.474	0.528	**0.945**	0.501	0.419	0.524
GNN	GCUNet (ours)	**0.894**	**0.493**	**0.548**	**0.555**	0.933	**0.564**	**0.544**	**0.621**

The bold values represent the highest scores. BG: Background; E-TLS: Early tertiary lymphoid structure; PFL-TLS: Primary follicle-like tertiary lymphoid structure; SFL-TLS: Secondary follicle-like tertiary lymphoid structure

### Visualisation

**Fig. 3:**
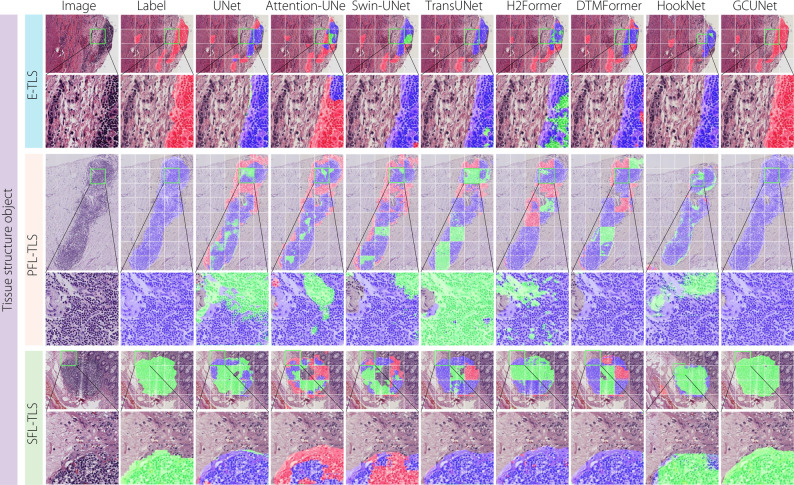
Visualization of segmentation results for three types of TLS—E-TLS, PFL-TLS, and SFL-TLS. E-TLS are highlighted in red, PFL-TLS in blue, and SFL-TLS ingreen according to our annotation guidelines. Each TLS contains a pair of images intwo rows: the top row shows a global view, while the bottom row provides a detailedview of the highlighted region. TLS: Tertiary lymphoid structure; E-TLS: Early tertiary lymphoid structure; PFL-TLS: Primary follicle-like tertiary lymphoid structure; SFL-TLS: Secondary follicle-like tertiary lymphoid structure

Figure 3 shows that GCUNet produces segmentation results that more closely match the ground-truth annotations, particularly in capturing fine-grained details and distinguishing TLS maturity levels. Other models struggle to effectively utilize discriminative features because of their inability to fully leverage contextual information beyond the target patch, resulting in poor consistency in TLS segmentation. GCUNet consistently performed well across all TLS types, especially in SFL-TLS regions containing GCs, where it produced clearer and more accurate boundaries than the other methods.

### Computational complexity

To evaluate the computational efficiency and hardware requirements of GCUNet, the number of trainable parameters, peak GPU memory during training and inference, inference throughput, and average inference time per WSI across all methods were compared. All the measurements were obtained on a single NVIDIA GeForce RTX 4090 GPU with a batch size of 16. The inference time was averaged over the same set of 45 WSIs for all methods. The reported parameter counts include all the trainable components of each segmentation model. For GCUNet, this includes the TransUNet segmentation backbone, GCN-based contextual aggregation module, and DCFusion module. The frozen UNI encoder was not included because UNI was used offline only to pre-extract fixed patch-level embeddings and was not loaded into the segmentation training or inference graph. The reported training and inference memory values correspond to the peak GPU memory consumption. The inference time includes GCN aggregation, patch-level segmentation, and prediction stitching but excludes foreground detection, patch extraction, disk input/output, and offline UNI feature extraction. The same measurement protocol and WSI set were used for all compared methods.

As shown in Table 5, GCUNet contains 110.58 million trainable parameters, representing an increase of 5.26 million parameters over TransUNet. Peak training and inference memory increase by only 0.20 GB and 0.02 GB, respectively. The average inference time increases from 42.08 to 44.78 seconds per WSI, corresponding to an additional 2.70 seconds. These results indicate that graph-based contextual aggregation and DCFusion modules introduce limited computational overhead relative to the TransUNet baseline. Compared to HookNet, GCUNet requires less peak training memory and achieves a shorter average inference time per WSI. HookNet requires 63.10 seconds per WSI, whereas GCUNet requires 44.78 seconds under the same evaluation settings. Overall, the computational overhead introduced by GCUNet remains moderate while enabling explicit long-range contextual modeling across WSI patches.

**Table 5:** Comparison of computational complexity and inference efficiency

Method	Trainable	Peak	Peak	Speed	Inference time
	Params (M)	Train Mem (GB)	Infer Mem (GB)	(patches/s)	per WSI (s)
U-Net	31.11	4.66	1.86	125	29.06
Attention-UNet	7.98	6.05	2.20	124	29.21
Swin-UNet	41.38	5.06	1.53	158	22.86
H2Former	33.70	11.99	2.78	87	41.84
DTMFormer	35.98	5.87	1.67	80	45.14
HookNet	15.93	9.93	2.11	57	63.10
TransUNet	105.32	9.16	1.95	86	42.08
GCUNet	110.58	9.36	1.97	81	44.78

Parameters denote the number of trainable parameters and exclude the frozen UNI encoder, which was used offline to pre-extract patch embeddings. Training and inference memory denote the peak GPU memory measured on a single NVIDIA GeForce RTX 4090 GPU. Inference efficiency was evaluated using the same set of 45 WSIs with a batch size of 16. The reported inference time includes graph construction, GCN-based contextual aggregation, patch-level segmentation, and prediction stitching, but excludes disk input/output and offline UNI feature extraction. WSI: Whole slide image; GCN: Graph convolutional network

### Ablation studies

Drawing on both quantitative and qualitative analyses, several factors influencing model performance were further investigated. These factors include the method of integrating detailed and contextual information, range of required contextual information, and information granularity of the patches.

**Fig. 4:**
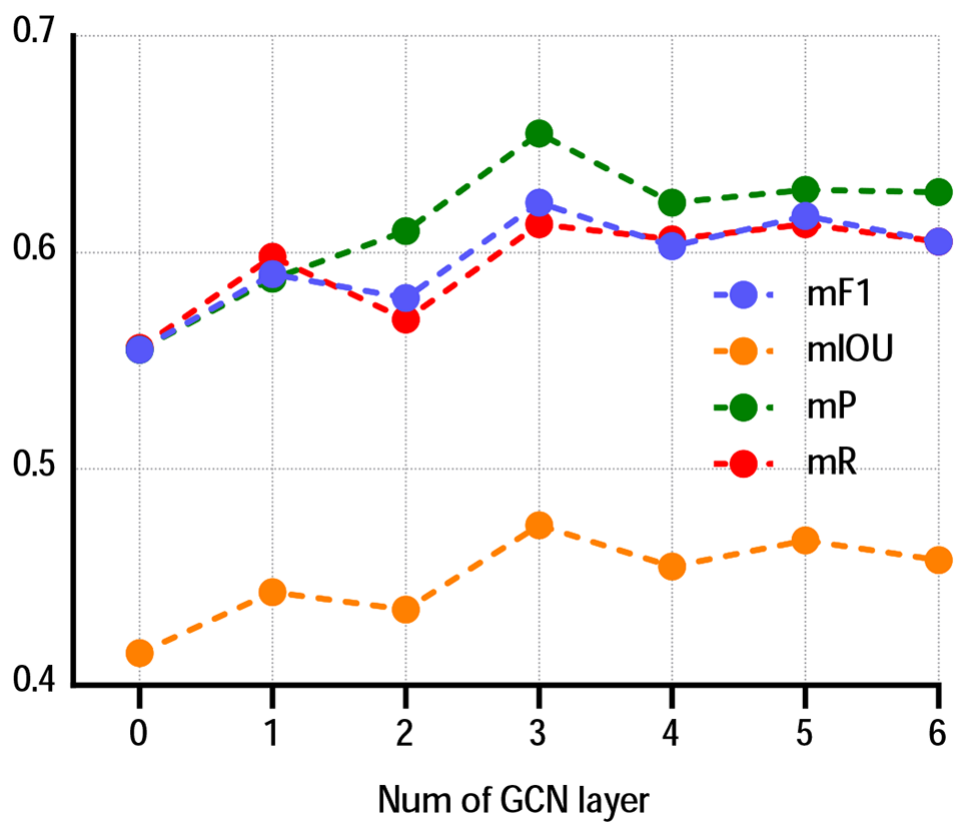
The impact of changing the number of graph convolutional network layers on four evaluation metrics. mF1: Mean F1-score; mIoU: Mean intersection over union

**Number of GCN layers**: Figure 4 presents the results of an ablation experiment exploring the effect of varying the number of GCN aggregation layers in Seg4 on the PUMCH-PAAD dataset. Let $\boldsymbol{N_c} = (0, 1, \dots, 6)$ represent the number of GCN layers, with a baseline corresponding to $\boldsymbol{N_c} = 0$. The number of GCN layers influences the extent of contextual information propagation for the target patch. $\boldsymbol{N_c}$ was iteratively adjusted and the corresponding segmentation performance was compared, keeping all other parameters constant. The figure shows that model performance is sensitive to the number of GCN layers. The segmentation performance decreased as the distance of the aggregated information was reduced, with the poorest performance observed when no contextual information was used. However, as the distance of the aggregated information increased further, the performance declined slightly and then stabilized. Across the evaluated GCN depths, the model achieved the best results when the number of GCN layers was set to 3.

**Fig. 5:**
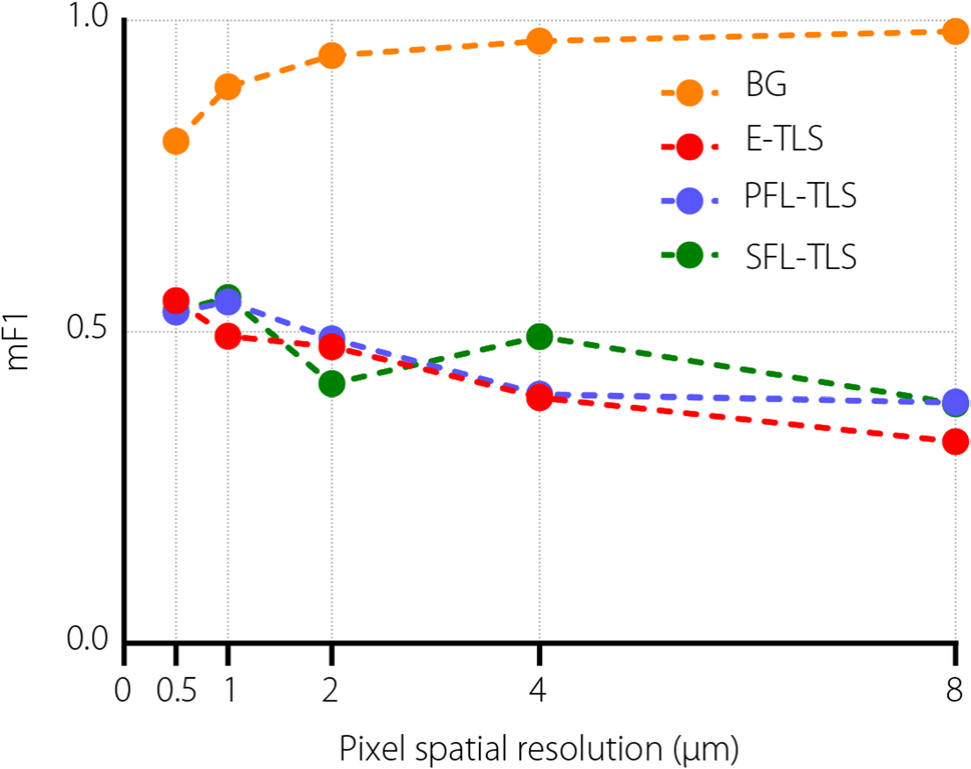
The impact of changing the pixel spatial resolution. BG: Background; E-TLS: Early tertiary lymphoid structure; PFL-TLS: Primary follicle-like tertiary lymphoid structure; SFL-TLS: Secondary follicle-like tertiary lymphoid structure

**Information granularity**: Figure 5 illustrates the impact of information granularity on the PUMCH-PAAD dataset. The number of GCN layers was set to three, the patch size to *224× 224*, and the pixel spatial resolution to $\boldsymbol{mpp} = (0.5, 1, 2, 4, 8)$. Figure 5 presents the mF1 scores of the segmentation results. As the pixel spacing increases and the spatial resolution becomes coarser, the model becomes better at distinguishing the background, but the performance on TLS decreases. At coarse spatial resolutions, the largest performance degradation is observed for the TLS categories, whereas the background performance remains relatively high. These experiments suggest that TLS semantic segmentation requires fine-grained information. At a spatial resolution of 1 *μ*m/pixel, the model achieved a relatively balanced performance across both the background and TLS categories.

**Context representation ablation:** To investigate the influence of different contextual feature representations on the segmentation performance, the contextual encoder was varied while keeping the TransUNet segmentation backbone unchanged. For all context-enabled configurations, the graph construction, number of GCN layers, and fusion strategy were kept identical. Four configurations were compared: (1) TransUNet without an external context, (2) GCUNet with a frozen ResNet-18 [44] contextual encoder, (3) GCUNet with a fine-tuned ResNet-18 contextual encoder, and (4) GCUNet with fixed UNI-based contextual features. Both ResNet-18 encoders were initialized using the same ImageNet-1K pretrained weights.

As shown in Table 6, the frozen ResNet-18 contextual encoder improves mF1 by 0.005 on PUMCH-PAAD and 0.004 on TCGA-COAD compared with the no-context baseline. Fine-tuning ResNet-18 increases the corresponding improvements to 0.037 and 0.072. These results demonstrate that the contextual-learning framework remains effective when a conventional CNN encoder is used instead of UNI.

**Table 6:** Ablation study on different contextual representations

Context setup	PUMCH-PAAD		TCGA-COAD	
	mF1	mIoU	mF1	mIoU
TransUNet (no context)	0.555	0.415	0.560	0.427
TransUNet + ResNet-18 (frozen)	0.560	0.421	0.564	0.431
TransUNet + ResNet-18 (fine-tuned)	0.592	0.449	0.632	0.492
TransUNet + UNI (GCUNet)	**0.623**	**0.474**	**0.665**	**0.523**

All methods use the same TransUNet backbone, while only the contextual encoder is varied. mF1: Mean F1-score; mIoU: Mean intersection over union

Replacing the fine-tuned ResNet-18 encoder with fixed UNI features further improves mF1 by 0.031 on PUMCH-PAAD and 0.033 on TCGA-COAD, while mIoU increases by 0.025 and 0.031, respectively. Relative to the no-context baseline, the UNI-based configuration improved mF1 by 0.068 and 0.105 for the two datasets. These findings indicate that UNI provides stronger pathology-specific contextual representations, whereas the improvements obtained with ResNet-18 show that the effectiveness of contextual learning is not solely dependent on UNI.

**Fusion strategy**: After determining the optimal resolution and number of GCN layers, experiments were conducted on various fusion methods to integrate the detailed and contextual information of the target using a pixel resolution of 1.0 *μ*m/px and three GCN layers on the PUMCH-PAAD and TCGA-COAD datasets. Several fusion methods were used: without contextual information (w/o-context), which does not incorporate contextual information and serves as the baseline for comparison; concatenation (Cat), which fuses target details and contextual information by concatenating them; dot product (Dot), which combines target details and contextual information using a dot product operation; and DCFusion, the fusion strategy of GCUNet, which employs a self-attention mechanism for semantic-level fusion of the two types of information. As shown in Table 7, all context-based fusion strategies outperformed the no-context baseline. Dot achieved higher performance than Cat on both datasets. DCFusion achieved the best performance on both datasets, improving mF1 by 0.068 and 0.105, respectively, compared to the no-context baseline. These results highlight the importance of semantic-level fusion of target details and contextual information.

**Table 7:** The impact of contextual information and detailed information fusion methods on model performance in the PUMCH-PAAD and TCGA-COAD datasets

Method	PUMCH-PAAD		TCGA-COAD	
	mF1	mIoU	mF1	mIoU
w/o-context	0.555	0.415	0.560	0.427
Cat	0.586	0.442	0.651	0.508
Dot	0.610	0.462	0.655	0.514
DCFusion (ours)	**0.623**	**0.474**	**0.665**	**0.523**

The bold values represent the highest scores. mF1: Mean F1-score; mIoU: Mean intersection over union

## Conclusions

In this study, a novel task of TLS semantic segmentation in WSI was introduced and a GCUNet was proposed. GCUNet uses GCNs to flexibly aggregate long-range and fine-grained contextual information beyond the target patch, while the designed DCFusion performs semantic-level fusion of detailed and contextual information to predict patch masks. Four TLS semantic segmentation datasets were gathered and annotations for three (TCGA-COAD, TCGA-LUSC, and TCGA-BLCA), comprising 826 WSIs and 15,276 TLSs, will be released. Results from these datasets showed that GCUNet achieved higher segmentation performance than the compared methods.

## Data Availability

The annotations generated in this study will be made available following publication.

## Abbreviation

TLS: Tertiary lymphoid structure
WSI: Whole slide image
E-TLS: Early tertiary lymphoid structure
PFL-TLS: Primary follicle-like tertiary lymphoid structure
SFL-TLS: Secondary follicle-like tertiary lymphoid structure
NSFL-TLS: Non-secondary follicle-like tertiary lymphoid structure
GC: Germinal center
GCN: Graph convolutional network
CNN: Convolutional neural network
GCUNet: GNN-based contextual learning network
DCFusion: Detail and context fusion
mF1: Mean F1-score
mIoU: Mean intersection over union
TCGA: The Cancer Genome Atlas
COAD: Colon adenocarcinoma
LUSC: Lung squamous cell carcinoma
BLCA: Bladder urothelial carcinoma
PAAD: Pancreatic adenocarcinoma
SOTA: State-of-the-art
mIF: Multiplex-immunofluorescence
CPath: Computational pathology
BG: Background
